# Social Distancing and Stigma: Association Between Compliance With Behavioral Recommendations, Risk Perception, and Stigmatizing Attitudes During the COVID-19 Outbreak

**DOI:** 10.3389/fpsyg.2020.01821

**Published:** 2020-08-11

**Authors:** Samuel Tomczyk, Maxi Rahn, Silke Schmidt

**Affiliations:** Department Health and Prevention, Institute of Psychology, University of Greifswald, Greifswald, Germany

**Keywords:** COVID-19, stigma, public health, risk communication, latent class analysis, infection prevention, cross-sectional

## Abstract

**Introduction:** Following behavioral recommendations is key to successful containment of the COVID-19 pandemic. Therefore, it is important to identify causes and patterns of non-compliance in the population to further optimize risk and health communication.

**Methods:** A total of 157 participants [80% female; mean age = 27.82 years (*SD* = 11.01)] were surveyed regarding their intention to comply with behavioral recommendations issued by the German government. Latent class analysis examined patterns of compliance, and subsequent multinomial logistic regression models tested sociodemographic (age, gender, country of origin, level of education, region, and number of persons per household) and psychosocial (knowledge about preventive behaviors, risk perception, stigmatizing attitudes) predictors.

**Results:** Three latent classes were identified: *high compliance* (25%) with all recommendations; *public compliance* (51%), with high compliance regarding public but not personal behaviors; and *low compliance* (24%) with most recommendations. Compared to high compliance, low compliance was associated with male gender [relative risk ratio (*RRR*) = 0.08 (0.01; 0.85)], younger age [*RRR* = 0.72 (0.57; 0.93)], and lower public stigma [*RRR* = 0.21 (0.05; 0.88)]. Low compliers were also younger than public compliers [*RRR* = 0.76 (0.59; 0.98)].

**Discussion:** With 25% of the sample reporting full compliance, and 51% differing in terms of public and personal compliance, these findings challenge the sustainability of strict regulatory measures. Moreover, young males were most likely to express low compliance, stressing the need for selective health promotion efforts. Finally, the positive association between public stigma and compliance points to potential othering effects of stigma during a pandemic, but further longitudinal research is required to examine its impact on health and social processes throughout the pandemic.

## Introduction

The current outbreak of the coronavirus SARS-CoV-2 and the associated disease, COVID-19, is transfixing the world with over 2 million confirmed infections by April 16, 2020^1^. In addition to its physical threat, this outbreak also causes psychological distress, anxiety, and depression (Wang et al., 2020). Moreover, research on the coronavirus-associated SARS pandemic in 2002/2003 points to potentially long-lasting adverse consequences, such as depression, stigmatization, diminished quality of life, and post-traumatic stress (Ko et al., 2006; Lee et al., 2006; Siu, 2008; Gardner and Moallef, 2015).

To contain infectious diseases like COVID-19, experts and government officials alike recommend a series of preventive behaviors, such as hand hygiene, and avoidance behaviors, such as social distancing or (voluntary) quarantine (e.g., Glass et al., 2006; Durham and Casman, 2012; Ding, 2014; Karimi et al., 2015; Weston et al., 2018; Lewnard and Lo, 2020). Previous simulations and current reports affirm that a combination of all strategies has the greatest success rates in containing the disease (Kelso et al., 2009; Kupferschmidt and Cohen, 2020). And yet, successful containment depends on adequate public compliance. While predictors of compliance can be explicated via a behavior theory (e.g., the theory of planned behavior; Ajzen, 1991), and they are well-documented for certain health behaviors (e.g., adherence in chronical illness; Rich et al., 2015), far less is known about compliance in pandemics.

To date, several studies have identified perceived personal risk (i.e., susceptibility, anticipated severity, and anticipatory worry) and knowledge of adaptive behaviors as facilitators of compliance (c. Tang and Wong, 2003, 2005; Cheng and Ng, 2006; Leppin and Aro, 2009; Kwok et al., 2020), although an explicit theoretical framework is often missing (Bish and Michie, 2010). Moreover, barriers to adherence (i.e., non-compliance) have received less attention presumably due to preventive and avoidance behaviors being very easy to carry out.

In a review of 26 studies on preventive behaviors in pandemics (Bish and Michie, 2010), however, compliance rates varied greatly, for example, between 4% for wearing a mask, 41.3% for “one or more specific actions” (Brug et al., 2004), and up to 95% for quarantine (Blendon et al., 2004). Despite the variety of illnesses, time frames, populations, and research methods in these studies, a general implication seems to be that a substantial proportion of the population does not adhere to the recommended behaviors. Composite measures of preventive behaviors revealed even lower compliance: 30.7% of a representative sample in Singapore practiced six or more out of eight (Quah and Hin-Peng, 2004), 48.7% in Hong Kong practiced five or more out of seven (Leung et al., 2003), and 37.8% in England practiced one or more out of three measures (Rubin et al., 2009).

In this respect, a qualitative study on (non)compliance with SARS quarantine identified ethical (e.g., civic duty), legal (e.g., monetary sanctions), and social (e.g., peer pressure) reasons to publicly comply with quarantine, while acceptance of quarantine differed markedly within households and private environments (Cava et al., 2005). Another study also identified practical issues (e.g., disposal of used tissues), selfishness, and responsibility shift (Morrison and Yardley, 2009) as core barriers to compliance. Responsibility shift refers to the belief that infected persons are particularly responsible for (not) spreading the illness, thus protecting others, whereas healthy persons are responsible for protecting themselves from becoming infected, leading to a shift in personal priorities in protective behaviors depending on one’s infection status.

Moreover, sociodemographic variables gender and age (i.e., male, younger age) consistently predicted non-compliance (Leung et al., 2003; Tang and Wong, 2003). This might be connected to a generally lower risk perception, particularly a lower perceived susceptibility, in young males (De Zwart et al., 2009). Regarding educational attainment, higher levels of education have been discussed as barriers to as well as facilitators of behavioral compliance in different populations (Leung et al., 2003; Tang and Wong, 2005; De Zwart et al., 2009; Bish and Michie, 2010).

To capture the existing heterogeneity in (non)compliance, this study utilizes a latent class approach (Collins and Lanza, 2010). Latent classes are often used to analyze behavioral patterns in non-communicable diseases, such as substance use (e.g., Tomczyk et al., 2015, 2016). However, to our knowledge, only one study applied latent class analysis to population behaviors following a novel virus outbreak [i.e., influenza A (H7N9)] in Hong Kong (Liao et al., 2015), despite the method’s statistical advantages in modeling behavioral patterns (e.g., flexibility, integration of measurement error). Liao et al. (2015) identified three latent classes of behavioral compliance, namely, moderate hygiene compliance (moderate personal hygiene, low avoidance behaviors), good hygiene compliance (high personal hygiene, low avoidance), and vigilance (high hygiene and avoidance). Moderate hygiene compliance was the largest class (about 50% of the sample) and was significantly associated with male gender, lower age, poor education, and lower risk perception, thus stressing the need for selective prevention and health promotion.

Finally, the current study also focuses on stigmatizing attitudes in the context of compliance due to the impact of stigma on fear, psychosocial stress, and social rejection during infectious diseases, such as SARS (Sim and Chua, 2004; Lee et al., 2005; Ko et al., 2006; Siu, 2008). Stigmatization can occur at different levels (e.g., individual, social, structural) and is connected to social identity processes (Tajfel and Turner, 1986; Bandura, 1998, 2004; Link and Phelan, 2001), where in-groups (i.e., individuals or groups that a person identifies with) and out-groups (i.e., individuals or groups a person does not identify with) are constructed based on certain characteristics (e.g., profession, illness symptoms). Out-groups are subsequently devaluated, for instance, by being labeled irresponsible or dangerous. This devaluation can further lead to verbal discrimination or interpersonal violence (Parker and Aggleton, 2003; Corrigan et al., 2004). Moreover, public stigma comprises support for a restriction of public opportunities (e.g., vote, utilize health care) for the devaluated out-group, in this instance, symptomatic and/or infected persons. In fact, survivors of the SARS epidemic experienced blame and social rejection (Lee et al., 2005; Mak et al., 2006), while persons of Asian descent reported victimization, regardless of their personal infection status (Zheng et al., 2005). These experiences of being blamed and ostracized oftentimes outlasted the epidemic and were associated with continued psychosocial stress (Brug et al., 2004; Siu, 2008; Jiang et al., 2009). In addition, an increase in influenza infections also corresponded to an increase in stigmatizing attitudes (e.g., a lack of trust, increased hostility) in previous research (Williams and Gonzalez-Medina, 2011).

Furthermore, qualitative studies argue that anticipated stigma might even prohibit personal preventive behaviors during infectious diseases, such as wearing masks, to avoid future stigmatization (Siu, 2008; Jiang et al., 2009); this hypothesis is supported by cross-sectional, quantitative research (Leppin and Aro, 2009). Similarly, perceived differences in responsibility for personal (healthy persons) and public protection (infected persons) during a pandemic (Morrison and Yardley, 2009) might reinforce stigma-associated social identity processes and increase the salience of group differences.

In sum, stigmatization might differentially affect behavioral compliance. On the one hand, it might be beneficial from a prevention perspective by fostering social distancing toward and isolation of infected people, primarily by stigmatizing persons and defining them as a relevant out-group (so-called *othering*; see Deacon, 2006). On the other hand, it might reduce compliance with official recommendations among stigmatized and/or infected persons due to fear of social isolation, stress, or discrimination (Williams and Gonzalez-Medina, 2011; Smith and Hughes, 2014). Therefore, to investigate compliance and the role of stigmatization during pandemics, this exploratory study aims to:

1. Examine patterns of intentions to comply with behavioral recommendations to contain the COVID-19 pandemic in the German population via latent class analysis.
2. Inspect the role of stigma in non-compliance while considering sociodemographic differences, risk perception, and knowledge of adaptive behaviors.
3. Explore intercultural similarities and differences of compliance by focusing on the German population, whereas previous research mostly focused on Asian populations.

## Methods

### Sample

*Via* an online survey, a community sample of 157 German adults [80% female; M (SD)_*age*_ = 27.82 (11.01)] provided information about their knowledge of preventive measures, risk perception, intentions to comply with official behavioral recommendations and guidelines as well as their stigmatizing attitudes toward people suffering from COVID-19. Participants received gift vouchers (€5) as incentives. The survey was conducted via convenience sampling between March 13 and March 27 by placing online advertisements on social media, for instance, on Facebook. During this time, far-reaching social isolation measures were implemented in Germany, for instance, restricting public meetings to two people (except for households) and establishing guidelines for a safety distance of 1.5–2.0 m in public spaces. In addition, behavioral recommendations on personal hygiene and avoidance behaviors were repeatedly and consistently issued by the government. The study procedure included informed consent in alignment with the Declaration of Helsinki and received ethical approval by a local ethics committee (BB 169/18).

### Measures

Sociodemographic data comprised age, gender [1 (*female*), 2 (*male*)], country of origin [0 (*Germany*), 1 (*other*)], level of education [0 (*lower secondary education*), 1 (*higher secondary education*, i.e., university entry level), 2 (*tertiary education*, e.g., bachelor’s degree)], region [0 (*rural*, i.e., up to 100,000 inhabitants), 1 (*urban*, i.e., more than 100,000 inhabitants)], and number of persons in one’s household [continuous; recoded as 1 (*1*), 2 (*2*), 3 (*3 or more*)]. For analysis purposes, categorical variables were dummy-coded.

Measures of stigmatizing attitudes were adapted from previous research on mental health stigma, assessing support for discrimination (Schomerus et al., 2007, 2019) with three items (“Persons with COVID-19 should not be allowed to hold public office,” “Persons with COVID-19 should not be allowed to have a driver’s license,” “If persons with COVID-19 do not consent to medical treatment, they should receive compulsory treatment”), and blame (Corrigan et al., 2006; Schomerus et al., 2019) with four items (e.g., “Persons with COVID-19 are to blame for their problems”) rated on a five-point scale each, from 1 (*don’t agree at all*) to 5 (*agree completely*). Support for discrimination (Cronbach’s α = 0.71) and blame (α = 0.73) showed satisfactory internal consistency.

Risk perception comprised two items representing cognitive and affective aspects of perceived risk, namely, perceived susceptibility (“How likely will you become infected?”; 0 to 100%) and anticipated fear [“How afraid would you feel if you became infected?”; 1 (*not at all*) to 5 (*very*)].

Intentions to comply with official recommendations were assessed by asking participants how likely [1 (*not at all*) to 5 (*very*)] they would follow the following nine recommendations: (1) covering mouth and nose with flexed elbow or tissue when coughing or sneezing; (2) avoid handshakes; (3) avoid touching one’s face (i.e., eyes, nose, and mouth) as much as possible; (4) dispose of used tissue immediately and securely; (5) frequent ventilation; (6) increased hand hygiene; (7) stay at home when sick/symptomatic; (8) avoid personal contact to symptomatic persons; (9) avoid mass events. Since strictly following these recommendations is the safest way to contain further spreading of the infection, we recoded items to reflect likelihood of compliance [1 (*very high likelihood*), 0 (*other*)]. These nine indicators were then subjected to latent class analysis. In addition, a single item measuring subjective knowledge of adaptive behaviors was rated from 1 (*very low*) to 5 (*very high*). All measures are listed in Supplementary Table S1.

### Statistical Analysis

Following an inspection of missing data and descriptive data analysis, latent class models were computed to examine patterns of (non)compliance in the population. Subsequent multinomial logistic regression models inspected sociodemographic and psychosocial predictors of compliance patterns. Descriptive data analysis was performed with Stata 15.1 (StataCorp, 2017), and latent class models and multinomial logistic regression models were computed with Mplus 7.4 (Muthén and Muthén, 1998–2015). All analyses were based on α = 0.05.

We estimated latent class models of compliance via robust maximum likelihood estimation with 2,000 sets of random start values. The estimation process started with two latent classes (indicating full compliance and non-compliance), the number of latent classes was subsequently increased up to five, while comparing model fit between models. Model selection considered overall model fit, parameter sparseness, classification quality, and theoretical tenability (Nylund et al., 2007; Tomczyk et al., 2016, 2018). As an overall fit measure, the bootstrapped likelihood ratio test (BLRT) compared the estimated model to a model with one less class: a significant value indicated better fit of the current model. To achieve reliable estimates, we chose 50 random starts with 50 bootstrap draws for each comparison. The Akaike Information Criterion (AIC) and the sample size-adjusted Bayes Information Criterion (BIC) indicated sparseness of the model; a lower value meant a sparser model. Average latent class probabilities (AL) and entropy demonstrated classification quality that is the differentiation between latent classes. Values range between 0 and 1; the closer to 1, the better the fit; an entropy of at least 0.6 pointed to reliable estimates (Asparouhov and Muthén, 2014). Finally, latent classes needed to be interpreted based on the literature and theoretical background. Therefore, the best latent class solution was selected on statistical criteria as well as content validity.

Using the three-step approach (Asparouhov and Muthén, 2014), we calculated multinomial logistic regressions to predict compliance patterns by sociodemographic data and psychological variables (stigmatizing attitudes, risk perception, and subjective knowledge). For each regression model, relative risk ratios (RRRs) including 95% confidence intervals were reported as effect sizes.

## Results

### Descriptive Statistics

Missing data were low (37 missing values; 0.01% overall) and equally distributed among variables, suggesting missing at random. Therefore, complete cases were analyzed for descriptive statistics (Schafer, 1999; Dong and Peng, 2013), while full information maximum likelihood was used for latent class estimation. The sample was predominantly female, most persons did not have a migration background, and about a fifth lived in single households. Due to the very high level of education, the variable “education” was dichotomized for further analysis [1 (*tertiary*), 0 (*secondary*)]. Intentions to comply were mixed but particularly low for immediate disposal of used tissues, frequent ventilation, and reduced hand-to-face contact (Table 1).

**TABLE 1:** Overview of mean values and relative frequencies of sociodemographic data, risk perception, knowledge, intentions to comply with recommendations, and stigmatizing attitudes in a German community sample (complete cases with listwise deletion; *N* = 154–157).

**Variable**	***M* (*SD*) or *N* (%)**
Age (range: 18–77)	27.82 (11.01)
**Gender**	
Female	124 (80.0)
Male	31 (20.0)
**Level of education**	
Lower secondary	4 (2.6)
Higher secondary	91 (59.0)
Tertiary	59 (38.3)
**Region**	
Rural	105 (73.2)
Urban	42 (26.8)
**Country of origin**	
Germany	150 (95.5)
Other	7 (4.5)
**Persons in one’s household**	
One	30 (19.5)
Two	63 (38.9)
Three or more	61 (39.6)
Support for discrimination (range: 1–5)	2.50 (0.82)
Blame (range: 1–5)	1.42 (0.54)
Risk perception	
Susceptibility (range: 1–100%)	62.17 (20.27)
Fear (range: 1–5)	3.11 (1.05)
Subjective knowledge about adaptive behaviors (range: 1–5)	3.80 (0.76)
**Intentions to comply with behavioral recommendations (very high)**	
(1) Covering mouth and nose when coughing or sneezing	144 (91.7)
(2) Avoid handshakes	121 (77.6)
(3) Avoid touching one’s face as much as possible	28 (17.8)
(4) Dispose of used tissue immediately and securely	81 (52.3)
(5) Frequent ventilation	55 (35.3)
(6) Increased hand hygiene	113 (72.9)
(7) Stay at home when sick	128 (81.5)
(8) Avoid personal contact to symptomatic persons	124 (79.0)
(9) Avoid mass events	128 (81.5)

### Latent Class Models

Model fit criteria for latent class models are printed in Table 2. While entropy and information criteria were in favor of a model with four classes, the difference to a three-class model was only marginal (ΔAIC = 0.04; ΔSSABIC = 1.14), and according to the BLRT, the latter was preferable. Moreover, a fourth class would have been very small (*n* = 6; 4.8%) with similar conditional response probabilities to class 1 of the three-class model. Since it also showed good entropy and latent class separation (ALCP > 0.8) compared to the remaining models, the three-class model was chosen. The following descriptions of latent class counts and proportions are based on most likely latent class membership.

**TABLE 2:** Model fit criteria for latent class models of intentions to comply with behavioral recommendations regarding infection prevention in a German community sample (*N* = 157).

	**2 classes**	**3 classes**	**4 classes**	**5 classes**
Free parameters	19	29	39	149
BLRT	77.28***	29.01***	20.41	15.46
AIC	1423.81	1414.80	**1414.76**	1419.42
SSABIC	1421.74	1411.64	**1410.50**	1414.07
Entropy	0.60	0.70	**0.74**	0.74
ALCP	0.89	0.86	1.00	0.85
	0.88	0.81	0.82	0.77
		0.91	0.90	0.85
			0.80	1.00
				0.84

BLRT, bootstrapped likelihood ratio test; AIC, Akaike Information Criterion; SSABIC, sample size-adjusted Bayes Information Criterion; ALCP, average latent class probabilities. ***p < 0.001; fit criteria indicating the best model are printed in bold.

**FIGURE 1:**
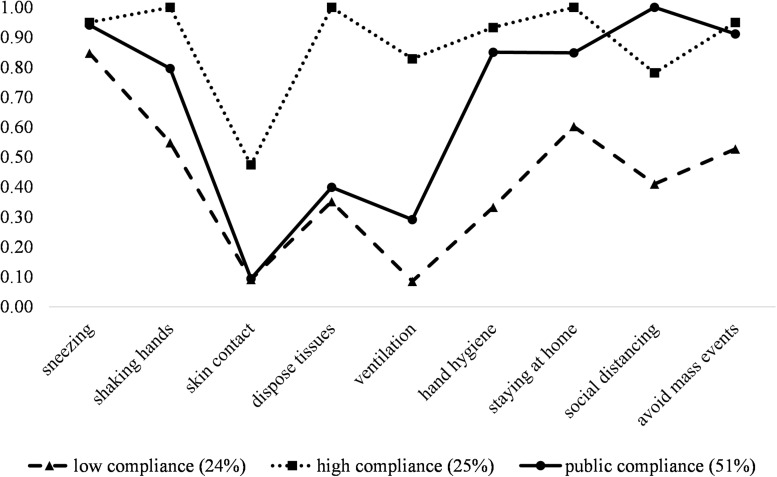
Conditional response probabilities and latent class proportions of three latent classes of (non)compliance with behavioral recommendations regarding infection prevention in a German community sample (*N* = 157). The probabilities correspond to the dichotomized likelihood of complying with recommendations [0 (*not at all likely* to *quite likely*); 1 (*very likely*)], thus a higher probability indicates higher compliance.

The first class was labeled “low compliance” (*n* = 37; 24%), with low to moderate intentions to comply with most recommendations except for covering one’s mouth and nose when sneezing or coughing. The second class was labeled “high compliance” (*n* = 40; 25%), with high probabilities of following most recommendations and moderate compliance with reducing hand-to-face contact. Finally, the third class, “public compliance” (*n* = 80; 51%), had high intentions regarding compliance with public and avoidance behaviors (e.g., social distancing) but low intentions regarding personal behaviors (i.e., avoidance of face contact, tissue disposal, frequent ventilation). Conditional response probabilities for each class can be seen in Figure 1.

Multinomial logistic regression compared sociodemographic data, stigmatizing attitudes, knowledge, and risk perception between latent classes (Table 3). To complement multinomial models, detailed descriptive comparisons of latent classes are provided in Supplementary Table S2. Compared to high compliance (class 2), low compliance (class 1) was associated with being male [*RRR* = 0.08 (0.01; 0.85)], younger [*RRR* = 0.72 (0.57; 0.93)], and expressing lower support for discrimination [*RRR* = 0.21 (0.05; 0.88)], whereas public compliance (class 3) and high compliance did not differ on sociodemographic data, stigmatizing attitudes or risk perception, although support for discrimination was considerably lower in public compliers than in high compliers [*RRR* = 0.27 (0.06; 1.21); *p* = 0.09]. Furthermore, low compliers were significantly younger [RRR = 0.76 (0.59; 0.98)] than public compliers and, by trend, were less fearful of a possible infection [RRR = 0.46 (0.20; 1.06); *p* = 0.07].

**TABLE 3:** Multinomial logistic regression of latent classes of intentions to comply with behavioral recommendations regarding infection prevention in a German community sample (*N* = 157).

**Predictor**	**Public compliance (class 3) vs. high compliance (class 2)**			**Low compliance (class 1) vs. high compliance (class 2)**			**Low compliance (class 1) vs. public compliance (class 3)**		
	**RRR**	**95% CI**		**RRR**	**95% CI**		**RRR**	**95% CI**	
Age	0.95	0.87	1.04	0.72*	0.57	0.93	0.76*	0.59	0.98
Gender (ref. male)	0.38	0.05	3.16	0.08*	0.01	0.85	0.22	0.02	1.90
Level of education (ref. secondary)	1.20	0.12	11.68	2.82	0.41	19.58	0.44	0.03	6.60
Region (ref. rural)	3.00	0.36	24.95	3.39	0.37	30.75	0.37	0.02	5.67
Country of origin (ref. Germany)	0.54	0.08	3.67	0.25	0.03	1.76	5.19	0.53	50.83
**Persons per household (ref. One)**									
Two	3.40	0.68	17.13	0.52	0.07	4.16	1.00	0.18	5.49
Three or more	0.15	0.01	4.12	1.11	0.03	41.84	1.60	0.02	119.22
Support for discrimination	0.27	0.06	1.21	0.21*	0.05	0.88	0.77	0.12	5.06
Blame	0.94	0.24	3.67	1.46	0.33	6.39	1.55	0.28	8.66
**Risk perception**									
Susceptibility	1.01	0.97	1.04	1.03	0.99	1.06	1.02	0.97	1.06
Fear	1.74	0.61	4.96	0.80	0.34	1.89	0.46	0.20	1.06
Subjective knowledge	0.46	0.13	1.67	0.25	0.05	1.26	0.55	0.08	3.84

RRR, relative risk ratio; 95% CI, 95% confidence interval. Significant coefficients are printed in bold; *p < 0.05.

## Discussion

As one of the first studies examining patterns of (non)compliance with behavioral recommendations in the general population during the COVID-19 pandemic, this study revealed that only a quarter of the surveyed German population expressed intentions to fully comply with recommendations, while a majority (about 51%) intended to follow some public actions but was less willing to enact personal hygiene behaviors (i.e., swift disposal of tissues, reduction of hand-to-face contact, ventilation). Young males were significantly less likely to comply with recommendations, and aspects of public stigma were also linked to compliance intentions.

In a virus outbreak, such as the COVID-19 pandemic, personal hygiene and social distancing in the general population are paramount to containment of the illness (Wu et al., 2006; Karimi et al., 2015; Weston et al., 2018). And yet, only a minority was ready to comply with the main recommendations, with 25% reaching high compliance in this sample and similar, albeit slightly higher, proportions of 30.7% (Quah and Hin-Peng, 2004), 37.8% (Williams and Gonzalez-Medina, 2011), and 48.7% (Lee et al., 2005) in previous studies. Since Germany was not affected by previous pandemics (e.g., H1N1, SARS) as strongly as Hong Kong, for instance, and measures like wearing face masks are not as common in Europe (e.g., Rubin et al., 2009), we assume the lack of familiarity with such strict preventive measures to be responsible for this lower level of compliance.

### Patterns and Predictors of Non-compliance

To further explore cultural differences of compliance during a pandemic and connect our findings to previous research, we compare our findings (Germany) to Liao et al. (2015), who analyzed latent classes of behavior patterns in Hong Kong during a virus outbreak. They also identified three latent classes, with the class *moderate hygiene* being the largest group, followed by *good hygiene* and *vigilance*. Moreover, younger males, persons with lower educational attainment, and lower risk perception were also more likely to belong to the moderate hygiene class (i.e., exhibit low compliance), similar to our findings. This trend of older persons and females reporting higher risk perception and willingness to perform preventive behaviors was consistently found in a variety of health risks (Flynn et al., 1994), among them also pandemics (Bish and Michie, 2010; Kwok et al., 2020), presumably due to a higher perceived susceptibility in these groups. Since older people have a higher risk of manifesting COVID-19 symptoms (Davies et al., 2020), which was promulgated via mass media reports, this might have led to lower susceptibility perceptions among younger people. Across cultures and scenarios, young males tend to report lower risk perception and compliance intentions. By corroborating these associations in the context of COVID-19, our findings stress the need for selective prevention targeting young males to improve their compliance and thereby public health.

Despite these similarities, we observed differing intentions regarding personal hygiene behaviors but overall high intentions to comply with avoidance behaviors, in contrast to Liao et al. (2015). While studies in other Western countries, that is, Canada (Toronto) and the United States (Blendon et al., 2004), also indicated high compliance with quarantine and social distancing strategies, it should be noted that avoidance measures are generally easier to implement than specific preventive behaviors that require personal action (Bish and Michie, 2010). Therefore, it is possible that in this early phase of the COVID-19 outbreak in Germany, personal responsibility was not as salient in the general population. This might be connected to the lack of familiarity with pandemics and appropriate preventive action in the German population. Nevertheless, personal preventive actions may yet increase over time, coinciding with an increase in vigilance, knowledge, and positive attitudes, if supported by concerted action, as suggested by previous SARS outbreak trajectories (Leung et al., 2003, 2005).

To concur, in their analysis of repeated cross-sectional surveys, Liao et al. (2015) observed fairly stable behavioral patterns (i.e., robust latent classes) across time but an increase in public vigilance and perceived threat throughout the epidemic (i.e., an increase in latent class proportions in favor of vigilance). To foster vigilance, the media and governmental institutions are therefore urged to provide clear guidance, openly communicate and justify new measures to increase trust, and strengthen self-efficacy at early stages of a pandemic, as shown in previous health crises (e.g., Seeger, 2006; Bean et al., 2015; Jha et al., 2018).

### Non-compliance and Stigmatizing Attitudes

In addition to compliance patterns, this study also examined the impact of stigmatizing attitudes on intentions to comply with behavioral recommendations. While Williams and Gonzalez-Medina (2011) connected an increase in influenza infections to an increase in stigmatizing attitudes, in this study, blame was low (mean = 1.42 on scale of 1–5) and did not predict compliance. Instead, support for discrimination was significantly associated with higher compliance intentions. Drawing on social psychiatric research, this type of discrimination might be described as *intentional structural discrimination*, where a worldview is actively supported that restricts patients’ rights (by law), for example, regarding their opportunities to vote or to hold public office (Corrigan et al., 2004, 2006; Schomerus et al., 2007). In the context of COVID-19, a support for discrimination implies a desired restriction of access to sociopolitical resources for infected persons.

As a result, while high compliance represents law-abiding and theoretically desirable behavior, its connection to discrimination, particularly in this highly educated sample, is noteworthy. In line with the reasoning behind selfishness and responsibility shift in confronting the SARS pandemic (Morrison and Yardley, 2009), a support for discrimination might indicate a way to maximize differences between relevant in-groups (i.e., responsible, healthy) and out-groups (i.e., irresponsible, reckless) to affirm social identity status (Tajfel and Turner, 1986; Link and Phelan, 2001) and – at least symbolically – reduce the risk of infection. Since blame did not differ between latent classes and was generally low, we assume that in this sample, stigma facilitated othering but not discriminatory action (Deacon, 2006). Although this hypothesis requires further research in larger, longitudinal samples using more elaborate measures of stigmatizing attitudes, it is clearly in line with evidence-based demands of a more nuanced debate of the functional properties of stigmatization and its connection to discrimination in infectious diseases (Deacon, 2006).

### Strengths and Limitations

Finally, this study is not without limitations, as the sample is a small convenience sample that is not representative of the German population. In fact, the sample was highly educated, predominantly female, and mostly without migration background. However, we still observed substantial heterogeneity in intentions, despite females and highly educated persons being generally more likely to report high compliance in previous studies. In addition, this study was cross-sectional and exploratory and used short but validated measures of core constructs, hence, effects of risk perception, for example, were not fully explored. Components like anticipatory worry could also affect compliance intentions and should be studied in more detail (Leppin and Aro, 2009). Furthermore, items measuring stigmatizing attitudes were adapted to COVID-19 for this study, therefore, a thorough psychometric validation is necessary. Moreover, we did not assess other important factors that might be connected to (non)compliance, such as ethnicity, interpersonal contact with infected persons, or trust in the government. Finally, we captured behavioral intentions, but we did not assess actual behaviors, as the pandemic had just reached the German population, and official recommendations were first issued at the beginning of data collection. Therefore, future studies should also focus on behavioral performance. When investigating the connection between compliance intentions and behavioral performance, health behaviors models like the theory of planned behavior should be applied to incorporate relevant intermediary variables, such as self-efficacy (Ajzen, 1991; Bish and Michie, 2010). Overall, more comprehensive, longitudinal, and experimental studies are necessary to validate our findings in the context of COVID-19 in diverse populations. Nevertheless, we think this study provides an important look at patterns of compliance at early stages of the COVID-19 outbreak and impactful sociodemographic and attitudinal factors, such as support for discrimination, that underline the need for selective preventive action.

## Data Availability Statement

The raw data supporting the conclusions of this article will be made available by the authors, without undue reservation, to any qualified researcher.

## Ethics Statement

The studies involving human participants were reviewed and approved by the Ethics Committee of the University Medicine Greifswald, University Medicine Greifswald. The patients/participants provided their written informed consent to participate in this study.

## Author Contributions

ST, MR, and SS contributed to the conception and design of the study. ST and MR were responsible for the data collection and statistical analysis. ST wrote the first draft of the manuscript. All authors contributed to the article and approved the submitted version.

## Conflict of Interest

The authors declare that the research was conducted in the absence of any commercial or financial relationships that could be construed as a potential conflict of interest.

## Footnotes

1 https://coronavirus.jhu.edu/
