# Supplementary material for: Social Distancing and Stigma: Association Between Compliance With Behavioral Recommendations, Risk Perception, and Stigmatizing Attitudes During the COVID-19 Outbreak
Source: Front Psychol. 2020 Aug 11;11:1821. doi: 10.3389/fpsyg.2020.01821 (PMC7432118; doi:10.3389/fpsyg.2020.01821)
Supplement: Supplementary file 1 [file Table_1.docx]

Supplementary Table S1

Measures and items used to analyze the association between compliance with behavioral recommen­dations, risk perception, and stigmatizing attitudes during the COVID-19 outbreak in a German community sample (items were translated from German to English)

| Construct | Items |
| --- | --- |
| Sociodemographic data |  |
| Age | How old are you?   - I am ____ years old. |
| Gender | What is your gender?   - Female - Male |
| Country of origin | Where were you born?   - Germany - Other country |
| Level of education | Please state your highest educational attainment.   - Hauptschule (lower secondary education; education in school years: 9 years) - Realschule (lower secondary education; education in school years: 10 years) - Abitur/Upper secondary education (university entry level) - University degree (i.e., tertiary education) |
| Region | Please estimate the number of inhabitants of the region you are currently living in.   - up to 100,000 - more than 100,000 |
| Number of persons in one’s household | How many persons live in your household (including you)?   - ____ persons |
| Stigmatizing attitudes |  |
| Support for discrimination | Please rate your agreement with the following statements.   - I do not agree at all (1) to I agree completely (5)   1. Persons with COVID-19 should not be allowed to hold public office.  2. Persons with COVID-19 should not be allowed to have a driver’s license.  3. If persons with COVID-19 do not consent to medical treatment, they should receive compulsory treatment. |
| Blame | Please rate your agreement with the following statements.   - I do not agree at all (1) to I agree completely (5)   1. Persons with COVID-19 are to blame for their problems.  2. COVID-19 infections usually result from a weak character.  3. Persons with COVID-19 only have to pull themselves together in order to get well.  4. One of the main causes of COVID-19 is lack of self-discipline. |
| Risk perception |  |
| Perceived susceptibility | How likely is it that you will become infected with COVID-19 in the future?   - 0% to 100% |
| Anticipated fear | How afraid would you feel if you became infected with COVID-19?   - not at all (1) to very (5) |
| Subjective knowledge of adaptive behavior | How much knowledge do you have about adaptive behaviors in case of a COVID-19 spread?   - very low (1) to very high (5) |
| Intentions to comply with recommendations | How likely is it that you would comply with the following recommendations?   - not at all (1) to very (5)   1. Covering mouth and nose with flexed elbow or tissue when coughing or sneezing  2. Avoid handshakes  3. Avoid touching one’s face (i.e., eyes, nose, and mouth) as much as possible  4. Dispose of used tissue immediately and securely  5. Frequent ventilation  6. Increased hand hygiene  7. Stay at home when sick/symptomatic  8. Avoid personal contact to symptomatic persons  9. Avoid mass events |
