# Supplementary material for: Social Distancing and Stigma: Association Between Compliance With Behavioral Recommendations, Risk Perception, and Stigmatizing Attitudes During the COVID-19 Outbreak
Source: Front Psychol. 2020 Aug 11;11:1821. doi: 10.3389/fpsyg.2020.01821 (PMC7432118; doi:10.3389/fpsyg.2020.01821)
Supplement: Supplementary file 2 [file Table_2.docx]

Supplementary Table S2

Mean values and relative frequencies of sociodemographic data, risk perception, knowledge, and stigmatizing attitudes in latent classes of intentions to comply with behavioral recommendations for COVID-19 prevention in a German community sample (complete cases with listwise deletion; N = 154-157)

|  | Class 1 (n = 37; 24%): Low compliance | Class 2 (n = 40; 25%): High compliance | Class 3 (n = 80; 51%): Public compliance |
| --- | --- | --- | --- |
| Age (range: 18-77) | 25.35 (7.82) | 30.95 (13.34) | 27.40 (10.77) |
| Gender  Female  Male | 25 (71.4)  10 (28.6) | 34 (85.0)  6 (15.0) | 65 (81.2)  15 (18.8) |
| Level of Education  Secondary  Tertiary | 24 (66.7)  12 (33.3) | 21 (52.5)  19 (47.5) | 50 (64.1)  28 (35.9) |
| Region  Rural  Urban | 19 (51.4)  12 (32.4) | 21 (52.5)  9 (22.5) | 36 (45.0)  21 (26.3) |
| Country of origin  Germany  Other | 35 (94.6)  2 (5.4) | 39 (97.5)  1 (2.5) | 76 (95.0)  4 (5.0) |
| Persons in one’s household  One  Two  Three or more | 8 (22.2)  12 (33.3)  16 (44.4) | 9 (25.0)  19 (47.5)  12 (33.3) | 13 (16.7)  32 (41.0)  33 (42.3) |
| Support for discrimination (range: 1-5) | 2.40 (0.76) | 2.73 (0.76) | 2.47 (0.82) |
| Blame (range: 1-5) | 1.49 (0.60) | 1.39 (0.52) | 1.41 (0.50) |
| Risk perception  Susceptibility (range: 1-100%)  Fear (range: 1-5) | 65.74 (14.52)  2.83 (1.00) | 62.08 (20.91)  3.23 (1.05) | 60.46 (22.28)  3.18 (1.07) |
| Subjective knowledge about adaptive behaviors (range: 1-5) | 3.70 (0.85) | 3.98 (0.83) | 3.76 (0.68) |
